# Longitudinal Mediation Modeling of Unhealthy Behaviors as Mediators between Workplace Demands/Support and Depressive Symptoms

**DOI:** 10.1371/journal.pone.0169276

**Published:** 2016-12-30

**Authors:** Linda L. Magnusson Hanson, Paraskevi Peristera, Holendro Singh Chungkham, Hugo Westerlund

**Affiliations:** 1 Stress Research Institute, Stockholm University, Stockholm, Sweden; 2 Indian Statistical Institute, North East Centre, Tezpur, India; 3 Department of Clinical Neuroscience, Karolinska Institutet, Stockholm, Sweden; North Carolina State University, UNITED STATES

## Abstract

Lifestyle has been regarded as a key pathway through which adverse psychosocial working characteristics can give rise to long-term health problems. The purpose of this study was to estimate the indirect/mediated effect of health behaviors in the longitudinal work characteristics-depression relationship. The analyses were based on the Swedish Longitudinal Occupational Survey of Health, including 3706 working participants with repeat survey measures on four occasions (2008, 2010, 2012 and 2014). Psychosocial work characteristics including demands and social support were analyzed in relation to depressive symptoms. Autoregressive longitudinal mediation models using structural equation modeling were used to estimate the intermediate effects of unhealthy behaviors including current smoking, excessive alcohol consumption, unhealthy diet and physical inactivity. Both workplace demands and social support were related to later depressive symptoms. In bivariate models we found no significant paths from workplace demands to health behaviors, but two out of three significant time-specific paths from workplace support to excessive drinking and from excessive drinking to depressive symptoms. Social support was also associated with subsequent unhealthy diet, and one path from unhealthy diet to depressive symptoms was found. However, despite indications of certain longitudinal relationships between psychosocial working conditions and health behaviors as well as between health behaviors and depressive symptoms, no significant intermediate effects were found (p>0.05). We conclude that changes in unhealthy behaviors over a period of two years are unlikely to act as strong intermediaries in the longitudinal relationship between job demands and depressive symptoms and between social support and depressive symptoms.

## Introduction

A range of health behaviors are increasingly acknowledged as important for health, not least mental health [1]. It has consistently been shown that physical leisure time activity is beneficial for mental health including depressive symptoms [2], while physical inactivity may cause disease [3]. There are also indications that certain dietary patterns influence the development of depression [1]. A healthy diet (rich in fruits, vegetables, fish and whole grains) and a Mediterranean diet appear to be associated with less depressive symptoms while an unhealthy (western) diet appears to be associated with higher level of depressed mood/depressive symptoms [4, 5]. Moreover, alcohol and smoking have been linked to development of depression [6–8], although prospective associations between depression and unhealthy behaviors have also been observed [2, 8–11]. An unhealthy lifestyle may have a negative impact on mental health through multiple mechanisms involving neurotransmitter imbalances, hypothalamic–pituitary–adrenal (HPA) axis disturbances, dysregulated inflammatory pathways, increased oxidative and nitrosative damage, neuroprogression, and mitochondrial disturbances, although the precise underlying mechanisms are not well understood [1].

Some evidence has also been presented on associations between work stress and health behaviors. Most previous studies have investigated work characteristics according to the theoretical demand-control-support model that includes components of psychological demands, job control/decision latitude and social support [12, 13]. Especially the job strain hypothesis has been investigated, which suggests that high job demands in combination with low control at work may be unfavorable to health. Job strain was not related longitudinally to alcohol intake [14], or to likelihood of taking up or quitting smoking [15] in large meta-analyses of occupational cohort studies from Europe. However, job strain has been found to increase the risk for physical inactivity [16] and a lower likelihood of adopting an overall healthy lifestyle, i.e. being of normal weight, a non-smoker, drinking moderate amounts of alcohol, and being physically active [17]. A healthy lifestyle may also be associated with a lower likelihood of job strain at follow-up and thus no straightforward cause and effect relationship has been demonstrated [17].

Bidirectional associations have also been observed with regard to psychosocial work characteristics and depressive symptoms [18], but there is now support for a prospective association between psychosocial work characteristics related to the demand-control-support model and depressive symptoms [19]. Earlier research has also indicated that sleep disturbances may partly explain the longitudinal relationship between work demands and depression [18]. Another key pathway through which exposure to psychosocial working characteristics can give rise to long-term health impairments is through health behaviors [20], but empirical evidence based on studies with repeat measures of both exposure, potential mediators, and outcome is lacking. The present study sought to examine whether unhealthy behaviors are intermediaries in the longitudinal relationship between job demands, decision authority, and social support and depressive symptoms.

## Materials and Method

### Study population

The study population consisted of participants in the Swedish Longitudinal Occupational Survey of Health (SLOSH) study, a nationally representative longitudinal cohort survey focusing on work life participation, social situation, and health/wellbeing. SLOSH started in 2006 with a first follow-up of participants in the Swedish Work Environment Survey (SWES) 2003 (n = 9,214), containing individuals from the entire country stratified by county, citizenship, gainfully employed and 16–64 years of age at the time of enrolment into SWES. About two years later, all eligible SWES participants were followed-up by means of postal self-completion questionnaires, one addressed to people in work, i.e. those in gainful employment for at least 30% of full time, and one to people working less or who had left the labor force temporarily or permanently. In 2006, wave one, 5,985 (65%) responded [21, 22]. All SWES participants from 2003 who were still alive, with a known address in Sweden and who had not actively opted out, were again asked to fill in questionnaires in 2008, 2010, 2012 and 2014. In 2008, all participants in SWES 2005 who were still alive were added to the cohort and followed up. In total, the number of respondents was 11,441 (61% of all eligible) in 2008, 10,078 (57%) in 2010, 9,880 (57%) in 2012 and 8,757 (52%) in 2014. The present study is based on the participants who responded and were working at least 30% in all four waves 2008–2014, yielding an analytic sample of 3,706 respondents. Some characteristics of these participants are presented in Table 1. In comparison with those who did not respond to all four waves and those who were excluded because they worked less than 30% in any of the waves, the analytic sample had a higher proportion of women, older persons, and people with university education. In 2008, the prevalence of high demands and low social support did not differ between the analytic sample and all who responded and working 30% or more in 2008, but unhealthy behaviors and depressive symptoms indicative of major depression were somewhat more common among all who responded and were working 30% or more in 2008. The study was approved by the Regional Research Ethics Board in Stockholm and informed consent was obtained from all participants.

**Table 1:** Descriptive statistics of background variables, work characteristics, health behaviors, and depressive symptoms at baseline (*N* = 3,706).

Variables	Mean/%	Minimum	Maximum	S.D.
**Age(years)**^**a**^	47.6	20	67	8.9
**Sex**				
Male	42.5	-	-	-
Female	57.5	-	-	-
**Education**^**b**^				
Unskilled manual workers	0.9	-	-	-
Skilled manual workers	5.9	-	-	-
Assistant non-manual employees	44.4	-	-	-
Intermediate non-manual employees	7.0	-	-	-
Professionals or upper-level executives	40.2	-	-	-
Self-employed	1.6			
**Civil status**^**b**^				
Married/cohabiting	79.4	-	-	-
Single	20.6	-	-	-
**Job demands score** ^**a**^^**,**^ ^**b**^	2.6	1	4	0.6
**Workplace social support score** ^**a**^^**,**^ ^**b**^	3.1	1	4	0.5
**Current smoking**^**b**^				
No	91.7			
Yes	8.3			
**Excessive alcohol consumption**^**b**^				
No	94.8			
Yes	5.2			
**Unhealthy diet**^**b**^				
No	93.2			
Yes	6.8			
**Physical inactivity**^**b**^				
No	83.9			
Yes	16.1			
**Depressive symptom score**^**a**^^**,**^ ^**b**^	5.4	0	24	5.1

S.D., standard deviation.

^a^ Mean values

^b^ information missing: education n = 1, civil status n = 30, job demands n = 15, social support n = 34, current smoking n = 18, excessive alcohol consumption n = 285, unhealthy diet n = 36, physical inactivity n = 30, depressive symptoms n = 59

### Measures

The SLOSH questionnaires for people in work contained numerous questions about the psychosocial work environment, work organization, health, and health-related complaints. Demands, control and support at work were measured in all four waves by the Swedish version of the Demand-Control Questionnaire (DCQ) [23], a widely used questionnaire for measuring these dimensions of the demand-control-support model with satisfactory psychometric properties [24]. Demands at work were assessed with four out of five demand questions (working fast, too much effort, enough time, and conflicting demands) [25]. Social support was measured with five questions from the DCQ included all in four waves (calm and pleasant atmosphere, good spirit of unity, colleagues are there for me, people understand a bad day, get on well with my superiors). The composite scales ranged from one to four. Decision authority as an indicator of control was also measured but not considered for further analyses since it was not associated with future depressive symptoms in previous analyses. Four dichotomous indicators of unhealthy behaviors were also created for each wave. Current smoking was categorized into daily smoking versus occasional (sometimes) or no smoking. Excessive alcohol consumption was determined using the alcohol use disorders identification test (AUDIT) 2006–2008 and the Cut-Annoyed-Guilty-Eye Questionnaire (CAGE) 2010–2012. Men reporting drinking twenty-one or more units and women fourteen 14 or more units weekly or drinking six or more units per occasion at least weekly based on AUDIT, and men and women reporting at least two problem drinking behaviors according to CAGE, were regarded to have excessive alcohol consumption. Diet was divided into unhealthy (a lot of fat, sugar or fast food) versus healthy diet (varying/average diet, avoid fat and sugar or special diet) based on a single question. Physical activity was divided into a physically inactive (exercises very little or never) versus an active (exercises now and then or regularly) group. A composite indicator of unhealthy behaviors was also considered by summing up the respective health behaviors into a measure of none to four unhealthy behaviors. Depressive symptoms were measured with a brief subscale from the Hopkins Symptom Checklist (SCL-90), the SCL-CD_6_[26] which assesses perceptions of being troubled by: Feeling blue; Feeling no interest in things; Feeling lethargy or low in energy; Worrying too much about things; Blaming yourself for things; and Feeling everything is an effort. The six items represent core symptoms, selected based on principals of clinical validity. The scale has been validated and was found to have good psychometric properties and results have showed that the items are suitable to add into a composite score (ranging from 0 to 24) indicative of depression severity [26].

### Statistical analysis

The present study assessed potential mediation by unhealthy behaviors utilizing an autoregressive approach based on structural equation modeling in order to simultaneously allow for paths opposite to the traditional direction (i.e. reverse causality) to be estimated [27]. Depressive symptoms and work characteristics were fitted as latent variables to reduce measurement error. Models were fitted separately for demands and social support. First bivariate structural cross-lagged models were fitted allowing correlations between all constructs and the errors of individual items over time to account for consistency in item-specific variance [28]. The cross-lagged paths estimate the effect of one variable on the other, after controlling for the stability of the variables over time [29]. There was no indication of measurement variance. Nested models with constraints on the structural paths over time, however, indicated that the paths of interest varied considerably over time. These paths were therefore allowed to be freely estimated in the final models. If the bivariate models showed any significant paths from the specific work characteristic to a specific unhealthy behavior and from the unhealthy behavior to depressive symptoms we further fitted structural autoregressive mediation models. Based on these models we assessed both the total effect (the effect of work characteristics on depressive symptoms either directly or indirectly through health behaviors), the direct effect (the part of the exposure effect which was not mediated by health behaviors) and the indirect effect (the part of the exposure effect which was mediated by health behaviors). The indirect effect (mediated effect) of the work characteristics on depressive symptoms through the mediators (i.e. daily smoking, excessive alcohol consumption, unhealthy diet, and physical inactivity) was estimated by the product of coefficients method. This method multiplies all paths that start with work characteristics in wave two and end with depressive symptoms in wave five which pass through unhealthy behavior(s) at least once in an intermediate wave. All paths from work characteristics at wave one to depressive symptoms at wave four that did not pass through unhealthy behaviors were also multiplied to estimate the overall direct effect. See also Magnusson Hanson et al. 2014 for a graphical illustration [18]. The statistical significance of the effect was evaluated using Monte Carlo Simulation confidence interval with 20,000 draws [30]. The degree of mediation over the entire period from wave two to wave five was finally assessed as the ratio between the overall indirect effect and the overall total effect. The analyses were conducted using the *lavaan 5*.*13*[31] package in *R*[32] in the years 2014–2015. A robust weighted least squares estimator was used which also allows for dichotomous and ordinal variables [33]. Furthermore, to reduce the bias introduced by missing information we used full-information maximum likelihood (FIML) estimation [34, 35]. Model fit was assessed by the comparative fit index (CFI), and the root mean square error of approximation (RMSEA) [36]. For the final models also standardized estimates were calculated. Potential confounders of work stress and depressive symptoms relationships such as age and sex (register data), civil status (married/cohabiting or single) and education (unskilled manual workers, skilled manual workers, assistant non-manual employees, intermediate non-manual employees, professionals and upper-level executives, or self-employed) from baseline were finally included to test the robustness of the results. Sensitivity analyses on smoking and physical inactivity were also conducted based on different operationalizations of these variables. In these analyses, we instead assessed putative mediation through smoking including both daily and occasional smoking compared to no smoking, and on physical inactivity assessing putative mediation through irregular physical inactivity compared to irregular (never, very little, or now and then) physical activity.

## Results

Bivariate models were first fitted to examine whether there were cross-lagged relationships between the exposure of interest and the putative mediators, and between the putative mediators and depressive symptoms, which is a prerequisite for a causal pathway via unhealthy behaviors. The bivariate models gave no support for a lagged effect of job demands on any of the unhealthy behaviors. No mediation model was therefore fitted for demands.

**Fig 1:**
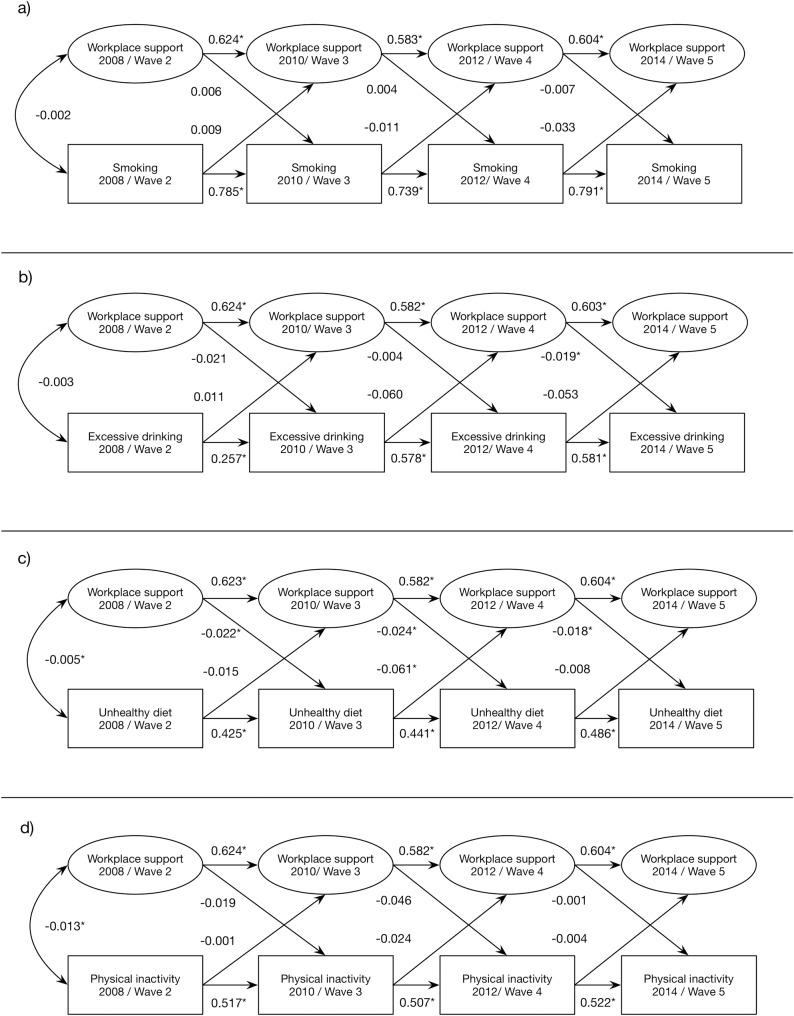
Standardized structural coefficients and p-values for the bivariate models including workplace social support and the respective unhealthy behaviors over four waves of the Swedish Longitudinal Occupational Survey of Health. * p<0.05

The bivariate models estimating the cross-lagged relationships between social support at work and the separate unhealthy behaviors showed that social support was not associated with subsequent smoking (Fig 1A), but two out of three time-specific paths showed a relationship between support and excessive alcohol consumption, see Fig 1B. A higher level of support was also related to a decreased risk of unhealthy diet two years later (Fig 1C). All paths from support to unhealthy diet were statistically different from 0 (β = -0.02), but no significant relationship between support and physical inactivity was noted (Fig 1D). All models were also adjusted for sex, age, civil status and education which improved model fit. As in the main analyses, the sensitivity analysis showed no association between social support and smoking when smoking was operationalized as any smoking instead of daily smoking. Workplace social support was on the other hand related to irregular physical inactivity in the sensitivity analysis, although only one of the three time specific paths was statistically significant (β = -0.04, p<0.05).

**Fig 2:**
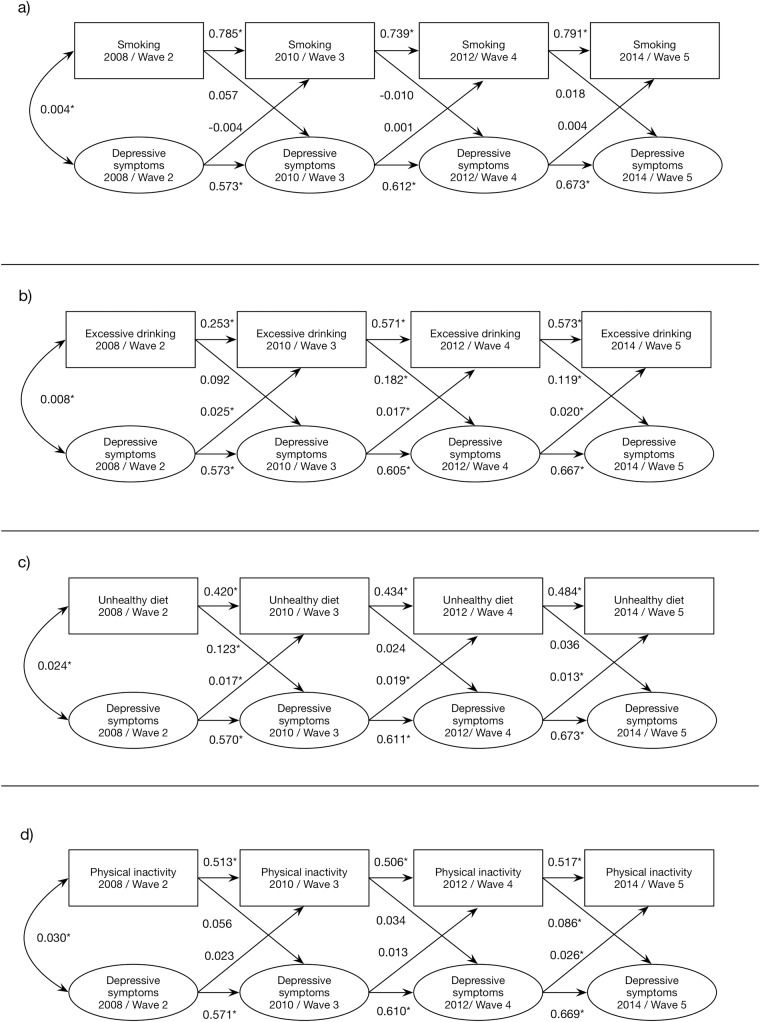
Standardized structural coefficients and p-values for the bivariate models including the respective unhealthy behaviors and depressive symptoms over four waves of the Swedish Longitudinal Occupational Survey of Health. * p<0.05

The bivariate models of the separate unhealthy behaviors and depressive symptoms showed that current smoking was not associated with later depressive symptoms, but we found some indications of a relationship between excessive alcohol consumption (two out of three significant time-specific paths), unhealthy diet (one time-specific significant path) and physical inactivity (one time-specific significant path) and depressive symptoms two years later, see Fig 2.

**Fig 3:**
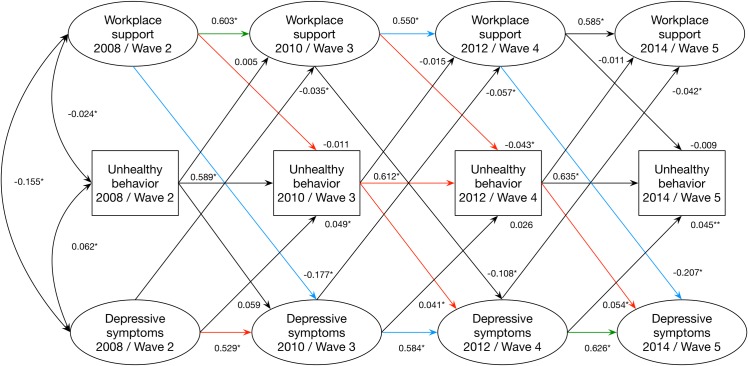
Standardized structural coefficients and p-values from the mediation model including workplace social support, number of unhealthy behaviors, and depressive symptoms over four waves of the Swedish Longitudinal Occupational Survey of Health. Red arrows represent paths that were used to calculate the indirect effect. Blue arrows represent paths that were used to calculate the direct effect. Green arrows represent paths that were used to calculate both the indirect and direct effect. * p<0.05 ** p<0.01

Taken together, the bivariate analyses regarding social support and unhealthy behaviors and between unhealthy behaviors and depressive symptoms suggested a potential mediating role for alcohol consumption, unhealthy diet, and physical inactivity (in sensitivity analysis). Mediation models were therefore fitted separately for these behaviors. These models supported an overall effect of support on depressive symptoms, a significant overall direct effect, but no intermediate effect of unhealthy behaviors (Table 2). The same was found in a sensitivity analyses assessing mediation via irregular physical inactivity. The same pattern was, furthermore, observed for the indicator of number of unhealthy behaviors (Fig 3).

**Table 2:** Total, direct and indirect effects calculated from the autoregressive mediation models. The standardized regression coefficients for the total effect were obtained by multiplying all paths in the models from social support to depressive symptoms, while the corresponding coefficients for the indirect effect were obtained by multiplying the paths from social support to depressive symptoms that passed through the mediator variable.

Model	Total effect(95% CI)	Direct effect(95% CI)	Indirect effect(95% CI)
Social support-Excessive alcohol consumption-Depressive symptoms	-0.10957 (-0.15040 to -0.06852)^a^	-0.10097 (-0.14980 to -0.06801)^a^	-0.00086 (-0.00310 to 0.00091)^b^
Social support-Unhealthy diet-Depressive symptoms	-0.10959 (-0.15150 to -0.06808)^a^	-0.10955 (-0.15010 to -0.06817)^a^	-0.00004 (-0.00320 to 0.00232)^b^
Social support-Physical inactivity-Depressive symptoms	-0.10962 (-0.15110 to -0.06901)^a^	-0.11085 (-0.17510 to -0.11180)^a^	-0.00123 (-0.00386 to 0.00110)^b^
Social support-Number of Unhealthy behaviors-Depressive symptoms	-0.11152 (-0.1504 to -0.06731)^a^	-0.10919 (-0.14990 to -0.06768)^a^	-0.00233 (-0.00282 to 0.00139)^b^

^a^ p<0.05

^b^ ns

With regard to the opposite direction, we found that excessive alcohol consumption in wave two was associated with job demands in wave three (β = 0.08, p<0.05), and unhealthy diet in wave three was associated with social support in wave four (β = -0.06, p<0.05), but these associations were not found between other waves (Fig 1). Depressive symptoms were more clearly associated with excessive drinking (β = 0.02) and unhealthy diet (β = 0.01 to 0.02) two years later (Fig 2). All models showed acceptable fit to the data.

## Discussion

The main aim of the present study was to investigate whether longitudinal associations between job demands and social support at work and depressive symptoms are mediated through unhealthy behaviors. The results did not support the hypothesis that unhealthy behaviors play a prominent role as intermediaries in a causal chain from these psychosocial work characteristics to depressive symptoms.

In line with Heikkila et al. 2012 neither job demands nor social support predicted the likelihood of taking up or quitting smoking [15]. Several previous prospective studies suggested a relationship between the job demand-control model and alcohol consumption or dependence [37]. Later studies have, however, showed inconsistent findings [38]. Inconclusive results were also found in the present study, but there were indications of a bivariate association between social support and drinking. A higher degree of social support appeared to be associated with a lower likelihood of excessive drinking. The most consistent finding in this study was, on the other hand, that workplace support was associated with a lower likelihood of unhealthy diet. Few studies thus far have addressed this relationship. Tamers et al 2011, however, also observed an association between worksite support and healthier dietary behaviors in terms of fruit and vegetable intake [39]. They also observed a relationship between worksite support and physical activity. However, we did not observe any clear relationship between job demands and physical inactivity or between support and physical inactivity. Nor did we find that depressive symptoms were prospectively related to physical inactivity, although some previous studies have suggested a longitudinal association also between job strain, job control and physical inactivity [16, 17, 40]. More knowledge seems warranted for the understanding of complex relationships between work stressors and unhealthy behaviors.

However, despite indications in the present study of bivariate associations between low social support and excessive drinking as well as unhealthy diet, no clear mediating pathway through any of the unhealthy behaviors was observed. We are only aware of one previous longitudinal study examining unhealthy behaviors as intermediate variables in the pathway from work characteristics to depression. That study found indications that the association between overtime work and ill health was partly due to unhealthy behaviors [41]. Overtime was particularly associated with physical inactivity and unhealthy diet. However, a limitation of that study is that it was only based on measurements from two time points and thus unable to test the temporal order between both overtime and physical inactivity as well as physical inactivity and depressive symptoms. At least three waves are needed to appropriately incorporate the temporal ordering of all three measures in modeling of mediation, which has been used in the present study [27]. The vast majority of studies on mediation have been cross-sectional and may therefore by biased [42]. In accordance with some earlier work, we also demonstrated that there may be bidirectional associations between psychosocial work characteristics and certain unhealthy behaviors as well as between certain unhealthy behaviors and depressive symptoms. In contrast to Taris et al. 2011 [41], we further studied and took into account bidirectional associations, which can improve our understanding of cause and effect.

However, it should be acknowledged that the results may be influenced by measurement error. Biased estimates of mediation may be observed especially when the mediator is measured with error. In this study, non-differential misclassification of the mediators may have attenuated our estimate of indirect effect and thus partly explain the lack of indirect effect. More detailed measurements of unhealthy behaviors would have been preferable to fully capture any associations through unhealthy behaviors. For example, under-reporting of alcohol consumption is a potential issue. Furthermore, we could not separate out alcohol abstainers which may be at increased risk for depression [43], study frequency or intensity of smoking, or detailed dietary patterns. Similar results were, however, found in sensitivity analyses on smoking and physical inactivity. A potential limitation of the study is also that the alcohol measure changed between the years 2008 and 2010, but roughly the same proportion of people were classified with excessive drinking in all waves. An advantage of the study is that missing information on the variables of interest was handled by full information maximum likelihood which is one of the best options for handling missing values in longitudinal data. The health behaviors were dichotomous rather than linear which pose a challenge to parameter estimation in structural equation modeling. To address violations to the normality assumption an alternative estimation procedure was used. For calculations of indirect effects standardized coefficients were further used which is recommended in the case of binary variables.

However, in order for a causal interpretation of the estimates of indirect and direct effects a number of additional assumptions need to be met. One assumption is that the models are correctly specified. There is a possibility that health behaviors may also moderate the relationship between work stressors and depression and if there is an interaction between exposure and mediator this should preferably be tested and included in the models. We were unable to appropriately test for this as models including interactions are not yet developed for a setting with time-varying exposure and mediators [44]. Based on the counterfactual framework in causal inference a set of definitions of direct and indirect effects have, however, been suggested that can be used for a more causal interpretation in situations with non-linearities and interactions. One is the natural indirect effect which assess the effect of the exposure on the outcome that operates by changing the mediator. Although statistical applications to obtain natural indirect effects are not yet well developed for longitudinal data, a randomized interventional analogue of the natural indirect effect has been proposed and can be calculated based on multiple waves of data using an autoregressive mediation modeling approach [44]. A randomized interventional analogue of the natural indirect effect of unhealthy behaviors based on three waves of data also pointed to a negligible indirect effect through the studied unhealthy behaviors, indicating that our estimates were not severely biased by violations to assumptions of nonlinearities and no interactions.

For a causal interpretation of the estimates of indirect and direct effects there should also be no omitted variables. This assumption may be violated if there is unmeasured confounding both of the exposure-outcome relationship, exposure-mediator relationship, and the mediator-outcome relationship. To reduce complexity we did not include potential time-varying confounders, including putative confounders for the mediator-outcome relationship, which may contribute to bias, but the results were not markedly different when adjusting for baseline sex, age, civil status and education. We also controlled for past values of the exposure, mediator, as well as outcome, which makes the no confounding assumption more plausible [44]. Other health behaviors may, however, be potential confounders of the mediator-outcome relationship and be affected by the exposure. By fitting separate models for the different unhealthy behaviors we have not been able to account for potential bias by other unhealthy behaviors. Although there are novel approaches in the causal inference literature for handling multiple mediators, they do not extend to situations in which the exposure and mediators are time-varying. A model including all the considered unhealthy behaviors simultaneously did, however, not lead to different conclusions.

A potential limitation is also that, the results may not be completely generalizable to the entire working population since it was based on those working and responding at all four waves, an analytic sample that was characterized by a high proportion of women, highly educated and older people with stable of relatively stable employment record. Dropout analyses indicated selective dropout only with regard to unhealthy behaviors and major depressive symptoms, but we cannot completely rule that dropout may have contributed to selection bias. Finally, when studying mediation, timing of the measurements in relation to the timing of cause and effect is critically important. If workplace stress only has a relatively immediate effect on health behaviors and/or unhealthy health behaviors are only associated with depressive symptoms in a short-time perspective we would have been unable to pick up a true indirect effect. However, relatively little is known about the appropriate lag between work stressors and unhealthy behaviors as well as between health behaviors and depression.

Given the limitations discussed above, the estimates provided here should not be interpreted as precise estimates of amount of exposure effect that operates through unhealthy behaviors. The results may, however, give an indication of whether change in unhealthy behaviors is a pathway through which job stressors can give rise to poor mental health. Although it is acknowledged that health behaviors are favorable to mental health, the present study did not support the hypothesis that the investigated unhealthy behaviors are important intermediate variables in the relationship between workplace demands and depressive symptoms or between workplace support and depressive symptoms, when considering a two-year time lag between the measurements. This suggests that interventions by way of health behaviors may not be very effective alternative to interventions targeting psychosocial work characteristics in preventing work-related long-term depressive symptoms. Future studies with more detailed and closely spaced measures are, however, needed to support or refute the results of this study and examine whether interventions to both psychosocial work characteristics and health behaviors may be more efficient than solely targeting the psychosocial work environment.
